# A Comparison Between Ultrasound-Guided Adductor Canal Block and Intra-articular Injection With Ropivacaine and Clonidine Combination for Postoperative Analgesia After Arthroscopic Knee Procedures: A Randomized, Double-Blind Clinical Study

**DOI:** 10.7759/cureus.86767

**Published:** 2025-06-25

**Authors:** Allwin Vino Prahasa, Anis Fathima, Nagalakshmi Palanisamy, Ranjan RV

**Affiliations:** 1 Anaesthesiology, Sree Mookambika Institute of Medical Sciences, Kanyakumari, IND; 2 Anaesthesiology, Pondicherry Institute of Medical Sciences, Pondicherry, IND; 3 Anaesthesiology, Sri Venkateshwara Medical College and Research Centre, Pondicherry, IND

**Keywords:** arthroscopy, artroscopic knee surgery, clonidine, postoperative analgesic, ropivacine

## Abstract

Background and objective

In recent years, arthroscopic procedures have gained significant popularity due to their distinct advantages, including minimal invasiveness, reduced postoperative scarring, quicker recovery, and lower surgical risks. However, inadequate postoperative pain control following orthopedic surgeries can hinder early mobilization, delay rehabilitation, and prolong hospital stays. The adductor canal block (ACB) has emerged as a favorable option for postoperative analgesia in such cases, offering benefits like ease of administration, cost-effectiveness, and compatibility with both general and spinal anesthesia. Likewise, intra-articular analgesia (IAA) is another effective method for managing postoperative pain. In this study, we compared the efficacy of ACB with intra-articular injection of a combination of ropivacaine and clonidine in patients undergoing arthroscopic procedures.

Methodology

Forty American Society of Anesthesiologists (ASA) physical status I/II adult patients were randomized into two groups (n=40). Group A received an ACB with 20 ml of 0.25% ropivacaine and 30 mcg clonidine, while Group B received an intra-articular injection with 20 ml of 0.25% ropivacaine with 30 mcg clonidine under ultrasound guidance after the ACB. Postoperative pain and duration of analgesia were assessed.

Results

Demographic and hemodynamic parameters were comparable between the two groups. However, there was a statistically significant difference in the time to first pain and the time to first rescue analgesia requirement. The mean time to first pain was 385.50 ± 44.90 minutes in the ACB group, compared to 311.00 ± 25.53 minutes in the IAA group (p<0.001). Similarly, the mean time to the first request for rescue analgesia was significantly longer in the ACB group (478.00 ± 36.22 minutes) compared to the IAA group (341.50 ± 24.12 minutes, p<0.001).

Conclusions

ACB using 0.25% ropivacaine combined with 30 mcg of clonidine provided superior postoperative analgesia compared to intra-articular injection with the same drug combination. It was associated with prolonged pain relief, a reduced need for rescue analgesia, earlier restoration of postoperative function, and maintained hemodynamic stability. The choice between these techniques can be individualized based on patient-specific factors, institutional guidelines, and availability of resources.

## Introduction

Arthroscopic knee surgeries, particularly anterior cruciate ligament (ACL) reconstruction, have become routine orthopedic procedures owing to their minimally invasive nature, reduced postoperative scarring, lower surgical morbidity, and quicker recovery time. Among the knee ligaments, the ACL is the most frequently injured, and arthroscopic ACL reconstruction is widely recognized as the gold standard for its management [1]. Optimal postoperative pain control is a cornerstone of successful outcomes in these surgeries, as it directly influences early mobilization, accelerated rehabilitation, and patient satisfaction [2]. Effective analgesia in the immediate postoperative period is essential to facilitate early range of motion and functional recovery of the knee. Inadequate pain control not only delays mobilization and discharge but also increases the risk of complications and healthcare costs [3]. While systemic opioid analgesics are effective, their use is limited by significant side effects such as respiratory depression, sedation, nausea, vomiting, and prolonged hospital stay. Consequently, the focus has shifted towards regional and multimodal analgesic strategies that offer effective pain control with fewer side effects [4].

Among the regional techniques available, adductor canal block (ACB) and intra-articular analgesia (IAA) are increasingly being employed for pain control following arthroscopic knee procedures. ACB, a peripheral nerve block, involves the ultrasound-guided administration of local anesthetic into the adductor canal - an aponeurotic tunnel located in the middle third of the thigh. This technique targets the saphenous nerve, a purely sensory branch of the femoral nerve, thereby providing selective sensory blockade of the anteromedial and medial aspects of the lower limb without compromising motor function [5,6]. Because of its ease of administration, safety, and motor-sparing characteristics, ACB has become a preferred option for postoperative pain relief in arthroscopic ACL repair. In contrast, IAA injection provides direct analgesia at the surgical site by delivering local anesthetics, opioids, or adjuvants into the joint space at the end of the procedure. Although IAA administration is simple and widely used, the effectiveness of local anesthetics alone in this route has been challenged, and the optimal combination of drugs and techniques remains an area of active research [7].

The combination of ropivacaine, a long-acting amide local anesthetic, and clonidine, an alpha-2 adrenergic agonist with analgesic and synergistic properties, has been shown to enhance postoperative analgesia when used in regional blocks and IAA injections [8]. Additionally, the use of ultrasound guidance has improved the precision and safety of regional blocks like ACB by allowing real-time visualisation of nerves, surrounding structures, and needle trajectory [9]. This has contributed to the increasing popularity of fascial plane blocks in orthopedic anesthesia. Given the importance of effective and opioid-sparing postoperative analgesia in arthroscopic knee procedures, a direct comparison between these two commonly used techniques (ACB and IAA) with a consistent drug combination is warranted. The current study is designed as a randomized, double-blind clinical trial to evaluate and compare the efficacy of ultrasound-guided ACB and IAA injection using a combination of ropivacaine and clonidine for postoperative pain control in patients undergoing arthroscopic knee surgeries.

## Materials and methods

Study design and ethical approval

This study was designed as a prospective, randomized, comparative clinical trial and conducted at the Pondicherry Institute of Medical Sciences, Pondicherry, India. Ethical clearance was obtained from the Institutional Ethics Committee of Pondicherry Institute of Medical Sciences (Ref no: RC/18/62), and the study was registered with the Clinical Trial Registry of India (CTRI/2019/03/018314). All participants provided written informed consent before enrollment, in accordance with ethical standards.

Sample size calculation

A total of 40 patients were randomized into two groups, with 20 patients in each group. The sample size calculation was based on the expected duration of the pain-free period (in minutes). A mean difference of 60 minutes between the two groups, with a standard deviation (SD) of ±55 minutes, based on previous literature [7]. With an alpha error of 0.05 and 80% power, a sample size of 40 patients (20 per group) was determined, accounting for potential dropouts.

Study population

The study included a total of 40 adult patients aged between 18 and 80 years, who were classified as American Society of Anesthesiologists (ASA) physical status I or II. These patients were scheduled for ACL reconstruction. Exclusion criteria were established with patient safety in mind and included the presence of local infection at the injection site, known allergy to local anesthetics, bleeding disorders, or an international normalized ratio (INR) above the normal reference range, and a BMI >35 kg/m^2^.

Preoperative preparation

All patients underwent standard preoperative fasting for at least six hours. Once in the operating room, baseline parameters including heart rate (HR), mean arterial pressure (MAP), and peripheral oxygen saturation (SpO₂) were measured. An 18-gauge IV cannula was placed, and IV fluid therapy was initiated using Ringer's lactate at a rate of 10 mL/kg/hour. With strict antisepsis precautions and using local anesthetic infiltration, subarachnoid block was performed by qualified anesthesiologists in L3- L4 space with 15 mg of 0.5% hyperbaric bupivacaine. A sensory level of T10 was achieved. Intraoperative HR, blood pressure (BP), respiratory rate, and SpO₂ were noted. If there was a failure of spinal anesthesia, general anesthesia was given, and the study was abandoned. Study drugs were prepared by an anaesthetist who was not involved in the study.

Randomization and group allocation 

**Figure 1:**
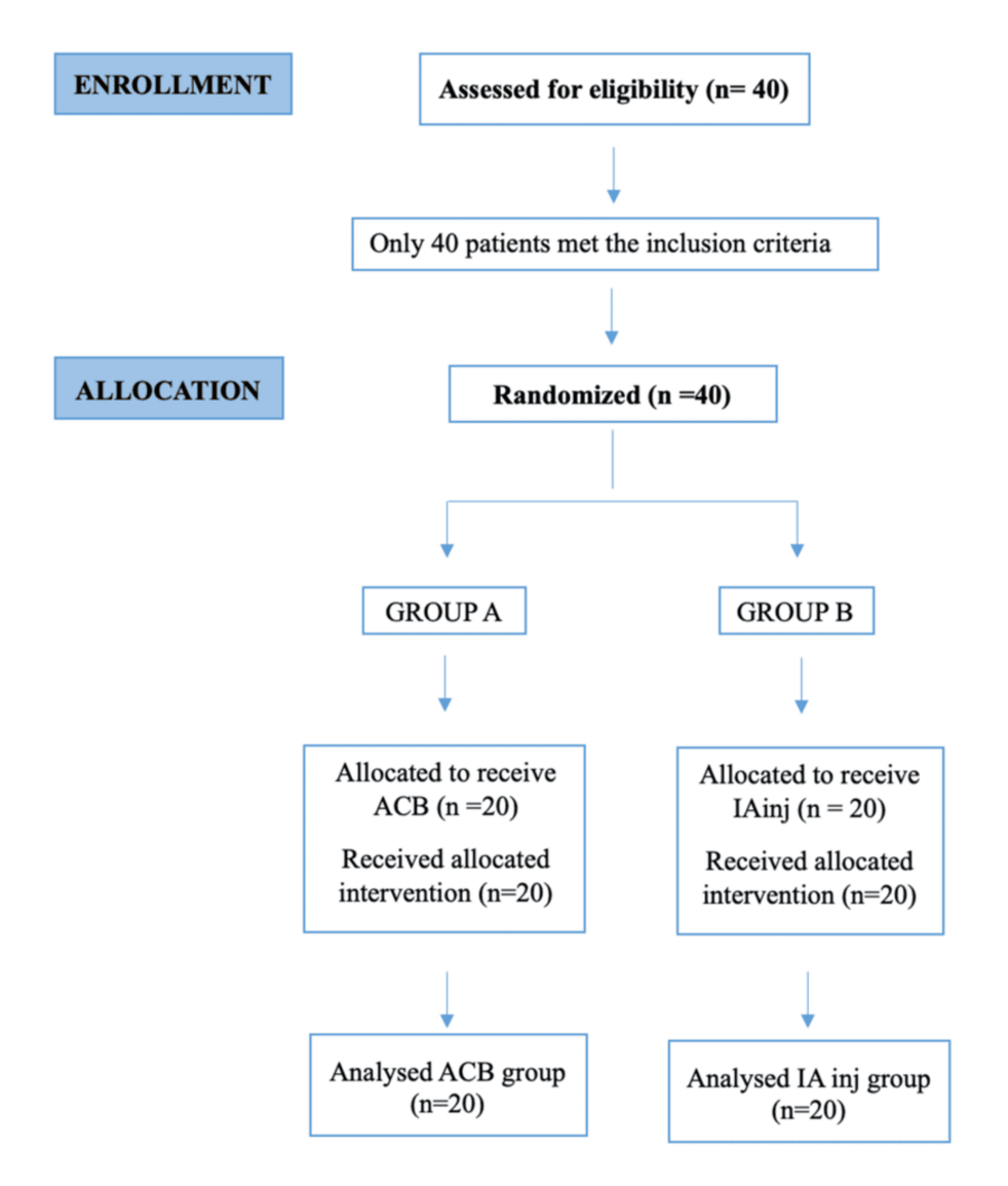
CONSORT flow diagram ACB: adductor canal block; CONSORT: Consolidated Standards of Reporting Trials; IA inj: intra-articular injection

Participants were randomized into two groups (20 each), based on a computer-generated block randomization chart. Group A received an ACB with 20 ml of Inj. 0.25% ropivacaine and 30 mcg clonidine. Group B received an intraarticular injection of 20 ml 0.25% ropivacaine and 30 mcg clonidine. The CONSORT (Consolidated Standards of Reporting Trials) diagram depicting patient selection and allocation is presented in Figure 1.

Techniques

Adductor Canal Block

In Group A, the ACB was performed under ultrasound guidance using a high-frequency linear probe (6-13 MHz). The transducer was placed transversely on the anteromedial thigh at the junction of the middle and distal thirds. The goal was to achieve the spread of local anesthetic lateral to the femoral artery and deep to the sartorius muscle. In the distal approach, the block was administered below the knee, adjacent to the saphenous vein. Using an 18-G venflon needle, 20 ml of 0.25% ropivacaine combined with 30 mcg of clonidine was injected in an in-plane approach. Additionally, 15 ml of normal saline was administered intra-articularly by the surgeon using a spinal needle before closure of the lateral arthroscopic port. Following the procedure, the portal was closed, a compression bandage was applied from the toes to the mid-thigh, and the tourniquet was released. 

Intra-articular Analgesia

In Group B, the surgeon administered 20 ml of 0.25% ropivacaine with 30 mcg of clonidine intra-articularly. A needle was inserted at the mid-thigh region, and a plaster dressing was applied to secure the site. After the administration of the block, the patient was kept under observation. 

Pain and hemodynamic monitoring

Sensory return around the knee joint was assessed at 30-minute intervals in the postoperative period until the first dose of rescue analgesia was given, at standardized anatomical landmarks just above and below the patella, avoiding areas covered by the dressing.. The duration of the sensory block was defined as the time from drug administration to the return of sensation around the knee joint. Hemodynamic and respiratory parameters, including HR, BP, respiratory rate, and SpO₂, were recorded at 30-minute intervals postoperatively until the administration of rescue analgesia. Following this, patients were monitored every two hours by the clinical staff. The first rescue analgesia (75 mg diclofenac intramuscularly) was administered when the visual analog scale (VAS) score exceeded 4 or when the patient first reported pain. The pain-free period was defined as the time interval between the administration of the study drug and the need for the first rescue analgesic. VAS scores were assessed every two hours, and data were recorded for total rescue analgesic consumption and the cumulative frequency of analgesia use over 24 hours for each group. Patients were transferred to the ward once they met the post-anesthesia care unit (PACU) discharge criteria. 

Statistical analysis

All data were analyzed using SPSS Statistics for Windows, Version 20.0 (IBM Corp., Armonk, NY). Continuous variables were summarized as mean ± SD or median with interquartile range (IQR), while categorical data were expressed as proportions and analyzed using the chi-square (χ²) test. Hemodynamic trends were evaluated using unpaired t-tests and validated through repeated measures ANOVA. Since VAS scores represent ordinal data, differences between groups at each time point were assessed using the Mann-Whitney U test. A p-value ≤0.05 was considered statistically significant in all analyses.

## Results

The baseline demographic characteristics, including age, height, and weight, were comparable between Group A (ACB) and Group B (IAA), indicating successful randomization. No statistically significant differences were observed in age or anthropometric parameters between the two groups. The mean weight in the ACB group was 71.45 ± 10.57 kg, while that in the IAA group was 69.90 ± 8.99 kg. Statistical analysis confirmed that this difference was not significant (p=0.620). Gender distribution and ASA physical status classification (ASA I vs. ASA II) were also evenly distributed across both groups, further supporting the homogeneity of the sample population (Table 1).

**Table 1:** Comparison of sociodemographic variables between the groups in the study (N=40) Fisher's exact test ACB: adductor canal block; ASA: American Society of Anesthesiologists; IA inj: intra-articular injection

Variables		Group		Total (40)	Chi-square test (p-value)
		ACB	IA inj		
Age group, years	20-30	8	4	12	0.192
	30-40	7	5	12	
	40-50	4	6	10	
	50-60	1	5	6	
Sex	Female	4	7	11	0.28
	Male	16	13	29	
ASA	1	17	19	36	0.605
	2	3	1	4	

In terms of the primary outcome relating to the duration of sensory analgesia, a statistically significant difference was noted between the two groups. Patients in the ACB group experienced a prolonged duration of sensory analgesia, with a mean of 385.50 ± 44.85 minutes, compared to 311.00 ± 25.53 minutes in the IAA group. This difference was highly significant, with a p-value of <0.001, indicating that the ACB technique provided superior analgesic duration. The timing of the first request for rescue analgesia was also evaluated. Patients in the ACB group requested rescue analgesia at a significantly later time (mean: 478.00 ± 36.22 minutes) compared to those in the IAA group (mean: 341.50 ± 24.12 minutes). This difference was statistically significant (p<0.001), reinforcing the prolonged analgesic benefit provided by ACB (Table 2).

**Table 2:** Association of duration of sensory analgesia with time of first request of rescue analgesia between the groups (N=40) Student’s unpaired t-test. P-value <0.05 is statistically significant ACB: adductor canal block; IA inj: intra-articular injection; SD: standard deviation

Variables	Group	Mean ± SD, minutes	P-value
Sensory analgesia	ACB (n=20)	385.50 ± 44.895	<0.001
	IA Inj (n=20)	311.00 ± 25.526	
Rescue analgesia	ACB (n=20)	478.00 ± 36.216	<0.001
	IA Inj (n=20)	341.50 ± 24.121	

Rescue analgesia requirements were also compared between the groups. In the ACB group, 18 patients required a single dose of rescue analgesia, while only two patients required a second dose. In contrast, in the IAA group, 11 patients needed one dose, and nine patients required two doses of rescue analgesia. This distribution showed a statistically significant difference (p=0.013), indicating a higher requirement for additional analgesia in the IAA group.

**Figure 2:**
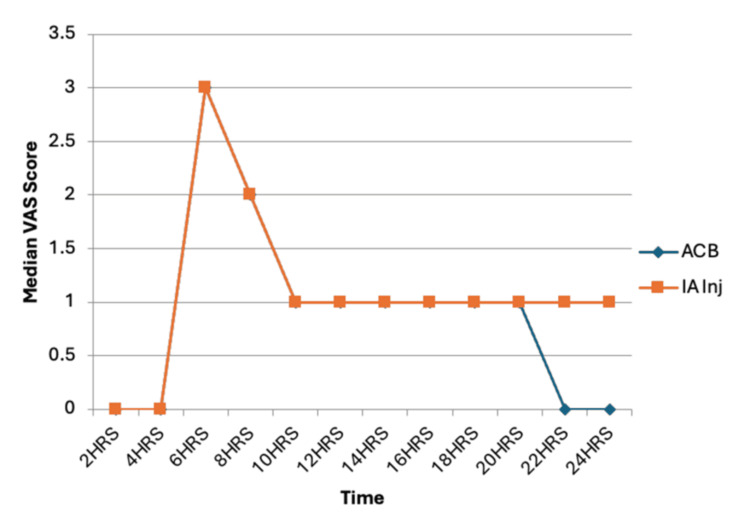
VAS score comparison between the groups ACB: adductor canal block; IA inj: intra-articular injection; VAS: visual analog scale

VAS scores were assessed postoperatively to evaluate pain severity. In both groups, the mean VAS score reached ≥3 by six hours postoperatively, necessitating administration of rescue analgesia (injection diclofenac). Following the administration of rescue analgesia, the VAS scores decreased to <3 in both groups. However, there was no statistically significant difference in VAS scores between the two groups at any measured time point (Figure 2).

**Figure 3:**
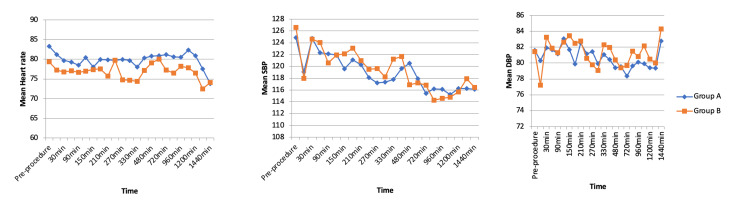
Comparison of nean HR, SBP, and DBP between the groups DBP: diastolic blood pressure; HR: heart rate; SBP: systolic blood pressure

Hemodynamic parameters, including HR, systolic BP (SBP), and diastolic BP (DBP), were monitored at various postoperative intervals. The HR ranged from 73.85 to 83.30 bpm in the ACB group and 72.45 to 80.00 bpm in the IAA group, with no statistically significant difference observed (p=0.052). Similarly, SBP ranged from 115.2 to 124.85 mmHg in the ACB group and 114.3 to 126.6 mmHg in the IA group (p=0.575). DBP ranged from 78.35 to 83.05 mmHg in the ACB group and 77.2 to 84.3 mmHg in the IA group (p=0.687). These findings indicate hemodynamic stability and comparable safety profiles in both analgesic techniques (Figure 3).

## Discussion

This randomized double-blind clinical study assessed and compared the effectiveness of ultrasound-guided ACB versus intra-articular injection of a ropivacaine-clonidine combination for postoperative analgesia in patients undergoing arthroscopic knee procedures. Forty ASA I-II patients aged 18-60 years were enrolled and randomly allocated into two equal groups. Pain relief was evaluated in terms of duration of sensory analgesia, time to first rescue analgesic request, number of rescue analgesic doses required, and hemodynamic stability over the 24-hour postoperative period. The study aimed to determine which technique offers more effective and sustained pain control with fewer side effects and better patient comfort.

Ropivacaine, a long-acting amide local anesthetic structurally similar to bupivacaine, has been widely used in orthopedic anesthesia. Its lower lipid solubility translates into reduced central nervous system and cardiac toxicity while maintaining effective analgesic properties. Samoladas et al. demonstrated that intra-articular ropivacaine significantly reduces postoperative pain and minimizes the requirement for systemic analgesics [10]. In our study, we used a combination of ropivacaine and clonidine via both ACB and intra-articular routes to compare their efficacy in patients undergoing arthroscopic knee procedures.

The demographic variables, such as age, weight, gender, and ASA classification, were comparable between the two groups, suggesting that randomization was effective in achieving baseline comparability. Although the mean age was slightly lower in the ACB group, this did not result in any statistically significant bias. The duration of sensory analgesia was significantly longer in the ACB group (385.50 ± 44.849 minutes) compared to the IAA group (311.00 ± 25.526 minutes), highlighting the superior analgesic efficacy of the ACB technique. This is consistent with the findings of Ibrahim et al., who reported an analgesic duration of 12.52 ± 1.16 hours with bupivacaine in ACB [11]. Arora et al. similarly found that the addition of clonidine to ropivacaine in ACB significantly prolonged pain-free periods (558.09 vs. 384.76 minutes) [12].

The time to first rescue analgesic request was also significantly longer in the ACB group (478.00 ± 36.216 minutes) compared to the IA group (341.50 ± 24.121 minutes), again demonstrating the sustained analgesic effect of ACB. These findings are supported by Kampitak et al., who reported a median time of 490.5 minutes for rescue analgesia in ACB patients [13]. Furthermore, fewer patients in the ACB group required multiple doses of rescue analgesia compared to the IA group. This is in agreement with Lefevre et al., who found a lower rescue analgesic requirement in patients receiving femoral nerve blocks (20%) compared to local infiltrative analgesia (39.1%) [14]. Zhao et al. conducted a meta-analysis comparing ACB and local infiltration analgesia in patients undergoing knee surgeries. The analysis included eight randomized controlled trials involving 675 patients. The study concluded that there was no statistically significant difference in postoperative pain scores between the two groups, suggesting comparable analgesic efficacy in certain clinical contexts [15]. Hemodynamic parameters remained stable throughout the study period in both groups. HR, SBP, and DBP variations were statistically insignificant between groups. These stable readings can be attributed to effective analgesia and the timely administration of rescue analgesics when the VAS exceeded 3. Arjun BK et al. also reported similar findings, noting that ACBs maintained hemodynamic stability without significant bradycardia or hypotension [16].

A key strength of this study is its randomized double-blind design, which minimizes bias and enhances the reliability of findings. The use of ultrasound guidance for ACB ensures precision in drug delivery and reflects modern clinical practice. The inclusion of a consistent drug combination (ropivacaine and clonidine) across both groups also allows for a focused comparison of technique efficacy rather than pharmacologic variability. However, this study has certain limitations. The sample size, while sufficient for detecting statistical differences in primary outcomes, was relatively small, potentially limiting the generalizability of the results. Additionally, the study only included ASA I and II patients aged 18-60 years undergoing elective arthroscopic knee surgery under spinal anesthesia, which may not represent all clinical scenarios, such as high-risk patients or those undergoing general anesthesia. 

The findings of this study reinforce the superior efficacy of ultrasound-guided ACB over intra-articular injection with ropivacaine and clonidine for postoperative pain relief following arthroscopic knee surgeries. ACB offers longer sensory analgesia, delays the need for rescue analgesics, and reduces analgesic consumption, without compromising hemodynamic stability. In this study, multimodal analgesia included the use of ACB or intra-articular injection combined with systemic non-opioid analgesics - specifically paracetamol and intramuscular diclofenac (75 mg) as rescue medication. Future research with larger and more diverse patient populations that incorporates long-term outcomes is recommended to further establish the clinical advantages of ACB.

## Conclusions

In this randomized double-blind clinical study, ultrasound-guided ACB using 0.25% ropivacaine with 30 mcg clonidine provided superior postoperative analgesia compared to intra-articular injection with the same combination. ACB resulted in prolonged sensory analgesia, reduced need for rescue analgesia, and enhanced functional recovery, without compromising hemodynamic stability. Given its efficacy, safety profile, and ability to facilitate early mobilization, ACB emerges as a preferred technique for postoperative pain management in arthroscopic knee surgeries. Future studies with larger sample sizes and long-term follow-up may further validate these findings and explore the role of ACB in enhanced recovery protocols.
